# *Aborjinia corallicola* sp. n., a new nematode species (Nematoda: Marimermithidae) associated with the bamboo coral *Acanella arbuscula* (Johnson)

**DOI:** 10.1007/s11230-021-09996-y

**Published:** 2021-08-14

**Authors:** Rickard Westerman, Bárbara de Moura Neves, Mohammed Ahmed, Oleksandr Holovachov

**Affiliations:** 1 Department of Zoology, Stockholm University, Stockholm, Sweden; 2 Ecological Sciences Section, Department of Fisheries and Oceans Canada, St. John’s, Newfoundland Canada; 3 Department of Zoology, Swedish Museum of Natural History, Stockholm, Sweden

## Abstract

A new species of *Aborjinia* Özdikmen, 2010 is described from the tissues of the cold-water bamboo coral *Acanella arbuscula* (Johnson) from the northwest Atlantic. *Aborjinia corallicola* **sp. n.** is characterized by 18.4–33.2 mm long body in adults; outer labial and cephalic sensilla papilliform and located 14–21 µm from anterior end, amphideal aperture located 22–41 µm from anterior end, excretory pore indistinct, rectum and anal opening functional, convex-conoid tail with broadly rounded terminus, spinneret subventral. It is placed in the genus *Aborjinia* based on the combination of the following characters: outer labial and cephalic sensilla papilliform in shape and located in one circle, contrary to *Marimermis* Rutsov & Platonova, 1974 (outer labial and cephalic sensilla setiform) and *Ananus* Rubtsov, 1977 and *Thalassonema* Ward, 1933 (outer labial and cephalic sensilla in separate circles, if known). From *Aborjinia eulagiscae* Tchesunov & Spiridonov, 1985 the new species differs in much shorter body, much shorter tail, presence of caudal glands and spinneret in adults, different host species*.* Our finding represents the first report of a nematode in a parasitic relationship with a cold-water octocoral. Phylogenetic relationships between *Aborjinia* and other nematodes are analyzed based on 18S rDNA sequences. Summary of all presently known species and genera of the family Marimermithidae is also given.

## Introduction

Marimermithidae Rubtsov & Platonova, 1974 is a small group of marine nematodes. They are widespread and have been recorded in south-western Indian Ocean, north and south Atlantic and in the north and south Pacific. They have been found at different depths all the way from the tidal zone, down to a depth of 5.2 km. Despite being so widespread, it is a rare group in which only seven species have been documented based on only a few specimens. Because of their rarity, not much of their life history is known, but it is believed to be similar to those in another rare group of marine parasitic nematodes of the order Benthimermithida. The marimermithids live as parasites during their juvenile stage and have been found parasitizing all kinds of aquatic invertebrates, for example echiuroids, priapulids, polychaetes, gastropods, sea urchins, starfish and basket stars. As adults they are free-living, and it is unknown if they feed at all during this stage, or if they like the Benthimermithids consume nutrients accumulated during the parasitizing stage (Miljutin, 2014).

Most nematodes are quite small, but the marimermithids stand out for their large size. Most of them measure between 5–10 mm. They have small non-spiral amphids. The anterior sensilla consists of six inner labial, six outer labial and four head sensilla in a ring formation. They lack a buccal cavity, their pharynx is simply built and the renette is distinguished with a very long channel. Numerous crystalloid bodies are found in the body cavity in some species. The intestine has distinct lumen, and the intestinal cells bear visible microvilli, while rectum and anus are almost always present. The main characters that distinguish this group from other groups of nematodes are their parasitic lifestyle, size, the crystalloid bodies in their body cavity and the hologonic ovaries filled with large amount of small eggs in mature females. It is still unclear where to place the marimermithids in the nematode classification system, since they share the majority of their characters with other groups of marine parasites, but also lack many features used in nematode classification (Miljutin, 2014).

The first species of Marimermithidae, *Thalassonema ophioctinis* Ward, 1933, was found by coincidence. It was during the investigation of the echinoderm fauna of South Africa when nematodes were found inside the brittle star of the species *Ophiocten amitinum* Lyman. The nematode was living in the brittle star's cavities and did not seem to harm the internal organs. Four ophiurans hosted eight nematodes, of which one female and five males were measured (the fate of the remaining specimens is not clear); they were comparatively large by nematode standards. Large males with a length between 11 to 16.5 mm and even larger females with a length around 22.8 mm had no distinct anterior sensilla on the anterior end; they also had no esophageal bulb or other specialized characters in their digestive tract. One character that distinguished these nematodes is the presence of granular cells in the perivisceral cavity. These cells occurred in both sexes and were believed to contain lipids used as storage for energy. Since the nematode did not seem to consume the brittle star and that the brittle star is eaten by many different bottom-feeding fish as well as other echinoderms like sea-urchins, it was assumed that the *Ophiocten amitinum* might be an intermediate host for this nematode (Ward, 1933).

Nearly forty years later, in 1974, Rubtsov and Platonova discovered and described two new genera of nematodes. These new nematodes were also parasites of marine invertebrates and the authors proposed a new family Marimermithidae for their finds. They named the new genera: *Marimermis* Rubtsov & Platonova, 1974 and *Trophomera* Rubtsov & Platonova, 1974. This new family included three genera: *Thalassonema* Ward, 1933, *Marimermis* and *Trophomera* and the genus *Marimermis* included three new species, *Marimermis maritima* Rubtsov & Platonova, 1974, *Marimermis kergelensis* Rubtsov & Platonova, 1974, and *Marimermis littoralis* Rubtsov & Platonova, 1974 (Rubtsov & Platonova, 1974). These new findings included six females of *Marimermis maritima* which were found at 10 m depth in the gravel off the coast of the Pacific side of Simushir Island, Kuril archipelago. The host of this nematode was originally not known (Rubtsov & Platonova, 1974), but later on another individual of the same species *Marimermis maritima* was found in the sea urchin *Strongylocentrotus polyacanthus* Agassiz & Clark collected near Kuril Islands in north-eastern Pacific (Tchesunov, 1995, 1997a). Kerguelen Island in the southern part of Indian Ocean hosted two discoveries. Four females of *Marimermis kergelensis* were discovered in a starfish (possibly *Hippasteria hyadesi* Perrier) at a depth of 214 m, while the last of those three *Marimermis* species, the *Marimermis littoralis* (one female), was found at the littoral of the Kerguelen Island; the host of this species is unknown.

Kerguelen Islands turned out to be a hot spot for marimermithid finds. In 1977, a new genus and species were found there (Rubtsov, 1977); five juveniles and females named *Ananus asteroideus* Rubtsov, 1977 were found in the starfish *Diplopteraster perigrinator* (Sladen). In 1985 a second species of the genus *Thalassonema,* the *Thalassonema ophiacanthis* Rubtsov, 1985, was discovered and described from the ophiuroid *Ophiacantha antarctica* Koehler from the southern part of the Atlantic Ocean, near the coast of Antarctica; just one immature male was found (Rubtsov, 1985). The main difference between *Thalassonema ophioctinis* and *Thalassonema ophiacanthis* was the size of the nematode body and size of spicules.

The same year a new genus and species were discovered and described: *Australonema eulagiscae* Tchesunov & Spiridonov, 1985 from the polychaete worm *Eulagisca gigantea* Monro from the Lazarev Sea near Antarctica. Three females were found and the main difference between *Australonema* and *Ananus* is in the arrangement of sensilla on the head (Tchesunov & Spiridonov, 1985). It must be noted that the name *Australonema* was already in use before Tchesunov and Spiridonov (1985) – Tassel (1980) used it to name a mollusk. According to the International Code of Zoological Nomenclature, a new name was proposed by Özdikmen (2010) – now these nematodes are called *Aborjinia* Özdikmen, 2010.

Because so much is unknown about marimermithids and so few species have been described, new species are frequently proposed for newly discovered populations. Several juveniles of unidentified *Australonema* found in the sea echiuroid collected in the Norwegian Sea have been studied by Tchesunov (1997b). The morphology of seven females and nine juveniles of another unidentified *Australonema* found in the polychaetes of the genus *Laetmonice* Kinberg from two localities in the Atlantic Ocean was also investigated and there are probably many more to find yet (Miljutin, 2003). Here we describe yet another marimermithid species *Aborjinia corallicola* **sp. n.** found associated with the cold-water bamboo coral *Acanella arbuscula* (Johnson) from the Grand Banks of Newfoundland, Labrador, and Davis Strait (Northwest Atlantic).

## Materials and Methods

**Fig. 1:**
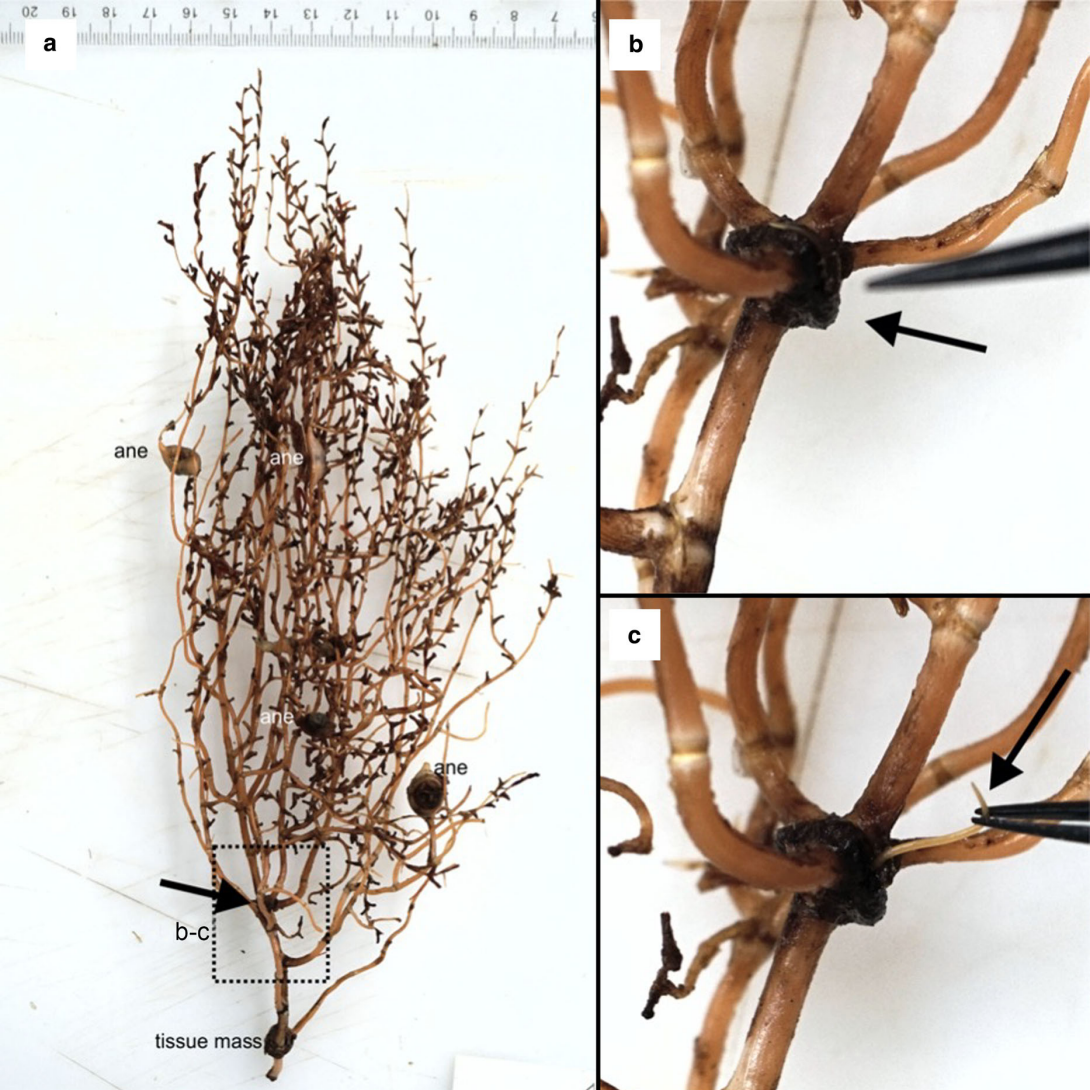
Photograph of a host coral *Acanella arbuscula*. a) shows the complete colony, where other associates can be identified (sea anemones, ane). Close-up of the hatched box is shown in b–c. b) tissue mass before nematode extraction (black arrow) and c) nematode being extracted from tissue mass (black arrow). Another tissue mass is seen at the base of the colony in a, which is missing its root-like branch. Inspection of this tissue mass did not yield any additional nematodes.

*Sampling and specimen preparation.* Nematodes were found during inspection of *Acanella arbuscula* colonies collected mostly during multispecies trawl surveys (HiBio-B-2019-A was collected using rock dredge) conducted by the Department of Fisheries and Oceans Canada (DFO) and additional material collected by fisheries observers in commercial fishing vessels (Table 1). Coral colonies were frozen at -20 °C immediately following collection. Nematodes were found underneath the tissue of the stem and branches in the *A. arbuscula* colonies (Fig. 1). Specimens were carefully removed from the corals and preserved in 100% ethanol. The number of specimens listed in Table 1 represents the total number of specimens found in each individual coral colony. A total of 110 nematodes were examined in this study, but not all specimens were preserved well enough for detailed morphological study. For light microscopy, specimens were transferred to pure glycerine using Seinhorst’s (1959) rapid method as modified by De Grisse (1969). Permanent nematode mounts on glass slides were prepared using the paraffin wax ring method. All curved structures were measured along the curved median line. Terminology follows Maggenti et al. (2005). Abbreviations are according to Hunt & Palomares-Ruis (2012). Specimens are deposited in the invertebrate collection of the Department of Zoology, Swedish Museum of Natural History, Stockholm, Sweden (SMNH) and in the Canadian National Collection of Insects, Arachnids and Nematodes.

**Table 1:**
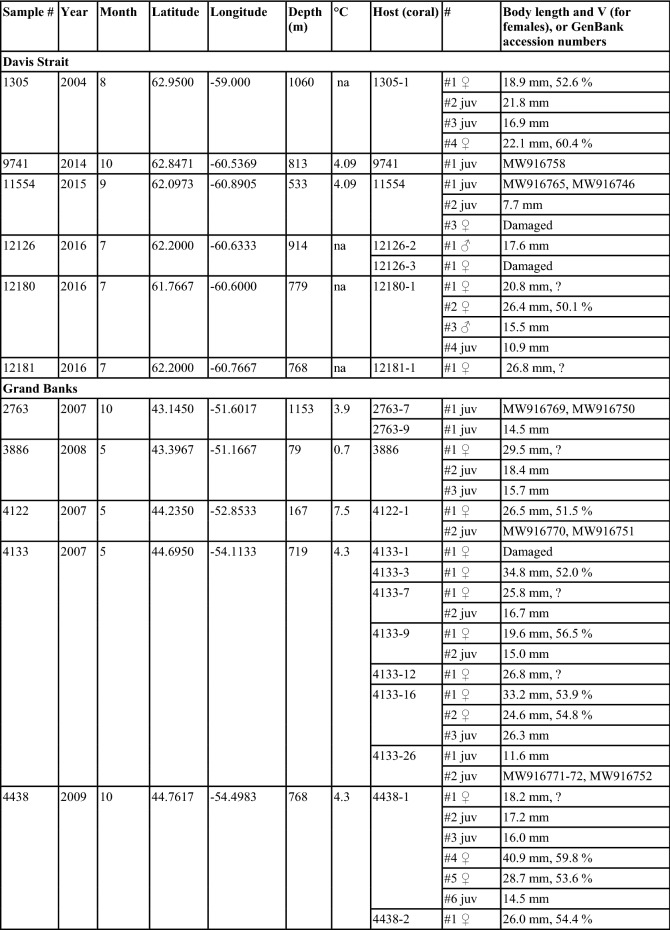

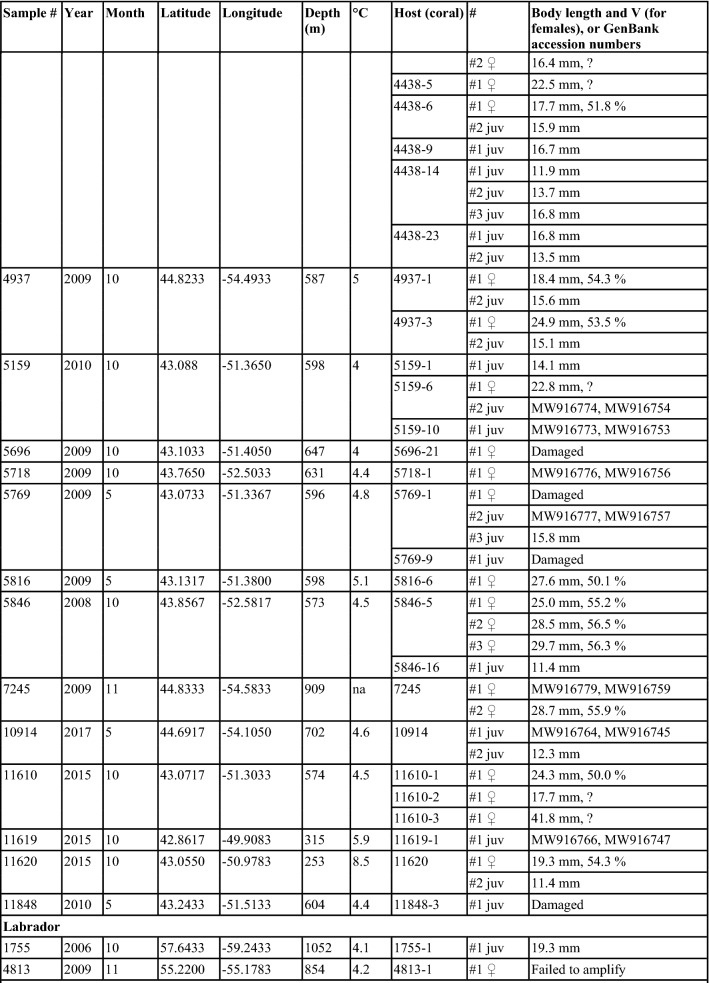

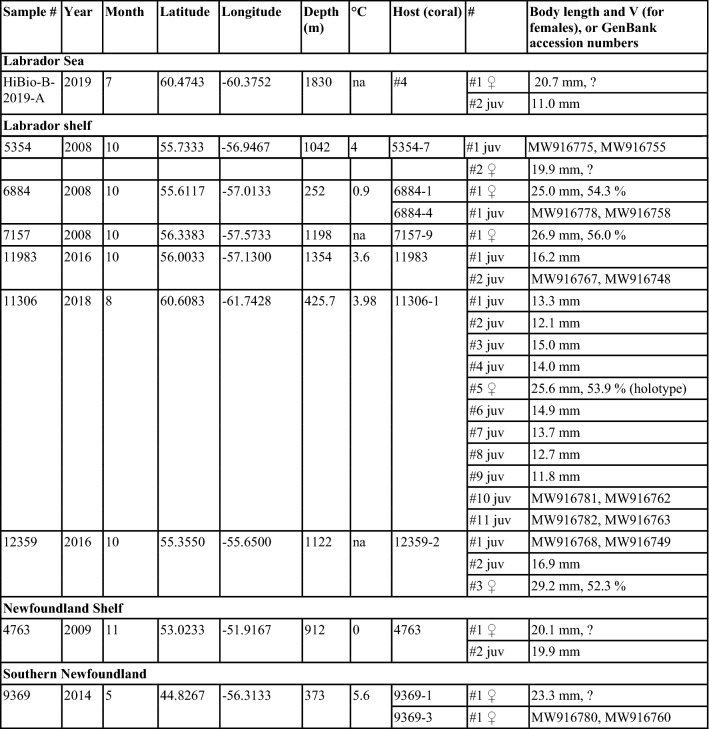
Sample data and basic information of all studied specimens (latitude and longitude in decimal degrees represent the start of a trawl set, and depth represents mean depth)

*Molecular analysis.* DNA extraction was performed on multiple specimens of *Aborjinia* from different localities (Table 1). Individual nematodes or middle sections of them were each placed in 1.5 ml microcentrifuge tubes containing 20 μl buffer ATL (Qiagen, Sweden) and stored at -20°C until all samples were ready for extraction. During the extraction, 160 μl of buffer ATL was added to each sample. This was followed by the addition of 20 μl proteinase K, vortexing and incubation in an incubating microplate shaker at 56°C and 300 rpm overnight. The lysed samples were further processed to obtain pure DNA following the manufacturer’s instructions for genomic DNA extraction using the Qiagen QiAmp DNA Micro kit. Two regions of the rRNA gene, the nearly full-length of the 18S and the D2–D3 expansion segment of 28S, were amplified. The approximately 1800 bp region of the 18S rRNA gene was amplified as two overlapping fragments using the primer sets 988F–1912R for the first fragment and 1813F–2646R for the second fragment (Holterman et al., 2006). Polymerase chain reaction (PCR) for both fragments was performed in 25 μl reaction mix using Illustra Hot Start Mix RTG 0.2 ml reaction kit (GE Healthcare Life Sciences, Sweden). The reaction mix consisted of 1 μl (0.4 μM) of each primer, 2 μl template DNA and 21 μl nuclease-free water. The reaction conditions were 5 min at 95°C; 5 cycles of (30 sec at 94°C, 30 sec at 45°C and 30 sec at 72°C); 35 cycles of (30 sec at 94°C, 30 sec at 54°C and 30 sec at 72°C); and a final extension for 5 min at 72°C. The D2–D3 segment of the 28S rRNA gene was amplified using the primers D2Af and D3Br (Nunn, 1992). PCR was performed in 25 μl reaction mix containing 1 μl (0.4 μM) of each primer, 2 μl template DNA and 21 μl nuclease-free water. The PCR conditions were 4 min at 94°C; 35 cycles of (94°C for 60 sec, 54°C for 90 sec and 72°C for 2 min); final extension for 10 min at 72°C. Enzymatic PCR clean-up was performed on the PCR product using Exonuclease I and Shrimp Alkaline Phosphatase (New England Biolabs, MA, USA). The purified PCR products were sent out to Macrogen Europe B.V. (Amsterdam, the Netherlands) for sequencing. Each amplicon was sequenced in both directions using the forward and reverse PCR primers. The trace files of the individual sequences were visualized inside BioEdit (Hall, 1999) and trimmed to high quality. The trimmed forward and reverse sequences were then assembled using Fragment Merger online tool (Bell & Kramvis, 2013). The two fragments of the 18S rRNA gene were also assembled into contigs using the Fragment Merger online tool.

*Phylogenetic analysis.* Alignment from Ahmed and Holovachov (2020) for 18S rRNA gene was used as template for alignment and annotation. New sequences were aligned to a fixed template alignment using AliView (Larsson, 2014). Phylogenetic trees were built using RAxML ver. HPC2 (Stamatakis, 2014) via the CIPRES portal (Miller et al., 2010) for the Maximum Likelihood inference of the partitioned dataset. The GTR nucleotide substitution model was used for non-paired sites, whereas the RNA7A (Higgs, 2000) substitution model was used for paired sites. Bootstrap ML analysis was performed using the rapid bootstrapping option with 1000 iterations.

## Marimermithidae Rubtsov & Platonova, 1974

***Aborjinia*** Özdikmen, 2010

*Type species*: *Aborjinia eulagiscae* (Tchesunov & Spiridonov, 1985) Özdikmen, 2010, by original designation [= *Australonema eulagiscae* Tchesunov & Spiridonov, 1985]

*Aborjinia corallicola* sp. n.

*Type host / Biology:* The nematodes were found in association with the bamboo coral *Acanella arbuscula*. This bamboo coral generally lives on soft sediments, to which it anchors through a calcareous root-like holdfast (Deichmann, 1936; Saucier et al., 2017). Nematodes were found underneath a layer of coral tissue covering their branches and stem, and sometimes inside a dark tissue mass involving branches (Fig. 1). Details on the occurrence rates of nematodes and other aspects of the association will be published separately (Neves et al. in prep). There are reports of nematodes found in epibiotic associations with other corals (Carvalho et al., 2014), or present in sediments or rubble associated with coral presence (Bianchelli et al., 2013; Cerrano et al., 2010; Pierrejean et al., 2020; Vanreusel et al., 2010), and the nematode *Deontostoma coptochilus* Hope, 1977 has been found in the foot cavity of the deep-water anemone *Actinauge longicornis* (Verrill) (Hope, 1977). However, to our knowledge the association between *Aborjinia corallicola* **sp. n.** and *A. arbuscula* represents the first report of an association between a parasitic nematode and a cold-water octocoral and likely other corals. For instance, in a review of invertebrates associated with cold-water corals, Buhl-Mortensen and Mortensen (2004) examined data on invertebrates reported from 74 deep-water coral species, and nematodes were not mentioned. Similarly, in other compilations of fauna associated with octocorals and black corals, nematodes were still not mentioned (Wagner et al., 2012; Watling et al., 2011).

Most of the nematodes examined were juveniles (54%), followed by adult females (45%) and only two adult males (Table 1). Juveniles, and adults (females and males) were found to often co-occur in the same coral colony, and more than one female was found in some colonies. Juveniles were not restricted to any month amongst the sampled months, being found in specimens collected in May, July, August-November.

**Fig. 2:**
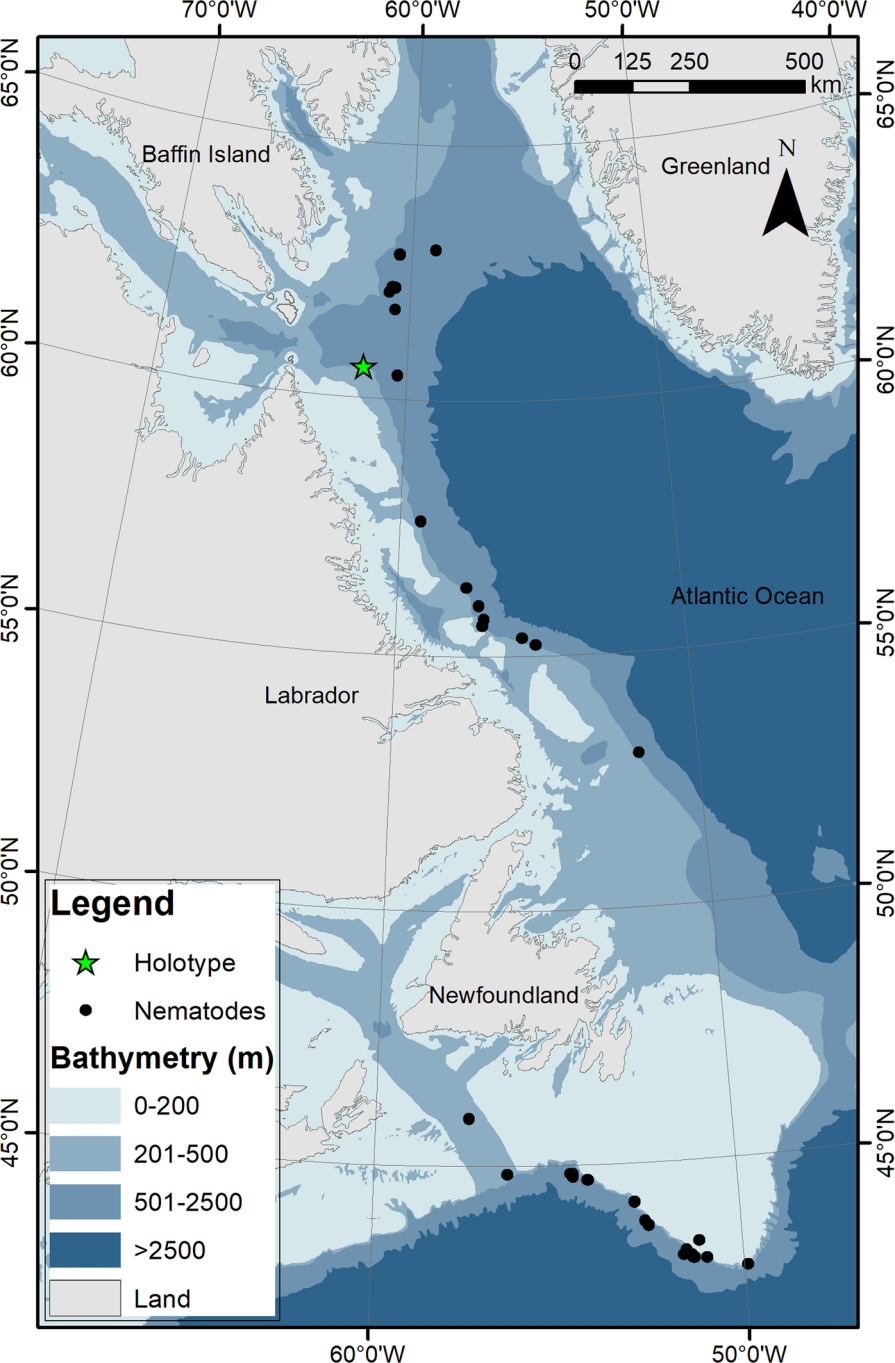
Distribution map of the specimens of the new *Aborjinia corallicola* **sp. n.** (and host) included in this study.

*Acanella arbuscula* is considered an indicator of Vulnerable Marine Ecosystems (Fuller et al., 2008) and this new finding further reinforces its role as habitat for other species, in this case for both juveniles and adults, and its potential role as an intermediate in nematode bioturbation. However, whether the nematodes utilize both the coral and the surrounding sediments is still unknown. No other nematode species were found in the examined colonies (N = 110 nematodes), which were collected from a wide latitudinal and bathymetric range (Fig. 2), indicating a potential specific relationship. *Acanella arbuscula* is also found in other regions of the world (Saucier et al., 2017), and inspection of colonies from outside the Northwest Atlantic might warrant additional records of the nematode. No other associates were found on the coral colony hosting the holotype.

*Type locality:* The holotype is from a coral colony collected in the Labrador Shelf, at 426 m in August 2018. The coral host was 9.5 cm x 8 cm (height x width), with a stem of 1.5 cm, and root-like holdfast measuring 2.9 cm in length. The colony was in good state of preservation, with a lot of tissue, and nematodes were found in a tissue pouch in the center of the colony. A total of 12 nematodes were found in this colony, which represents the maximum number found in a single colony, amongst the analyzed specimens.

*Other localities:* The nematode was also found in coral colonies collected along the Tail of the Grand Banks of Newfoundland, Labrador, and Davis Strait in the Northwest Atlantic, at mean depths ranging between 79–1830 m (Table 1, Fig. 2).

*Type material:* Holotype female on slide SMNH HT-9302 is deposited in the Invertebrate type collection of the Department of Zoology, Swedish Museum of Natural History, Stockholm, Sweden. Eight juvenile paratypes on two slides is deposited in the Canadian National Collection of Insects, Arachnids and Nematodes under the accession number T621.

*Other material:* Three females on slides SMNH 198157–198159 are deposited in the Invertebrate collection of the Department of Zoology, Swedish Museum of Natural History, Stockholm, Sweden. The remaining material on 68 slides is deposited in the Canadian National Collection of Insects, Arachnids and Nematodes under the accession number T621.

*Etymology:* The species name *corallicola* is composed of two parts: *corallum* (= coral) and *-cola* (= inhabitant, dweller), meaning "coral-dweller".

*ZooBank registration:* urn:lsid:zoobank.org:pub:67E52AD1-DC46-496E-957A-820D1F7B0ACC (publication); urn:lsid:zoobank.org:act:E6ECE3CE-372A-44AA-9EA6-F3FFFE09EE96 (species).

*GenBank acc. numbers:* Sequences obtained are deposited in GenBank under the accession numbers MW916745–MW916763 for the D2-D3 segment of the 28S rRNA gene and MW916764–5MW916782 for the nearly full-length 18S rRNA gene.

Description

*Diagnosis: Aborjinia corallicola* **sp. n.** is characterized by 14–42 mm long body in females and 15-18 mm in males; outer labial and cephalic sensilla papilliform and located 13–21 µm from anterior end, amphideal aperture located 22–41 µm from anterior end in females and 59–73 µm in males, excretory pore indistinct, rectum and anal opening functional, convex-conoid tail with broadly rounded terminus, spinneret subventral.

**Fig. 3:**
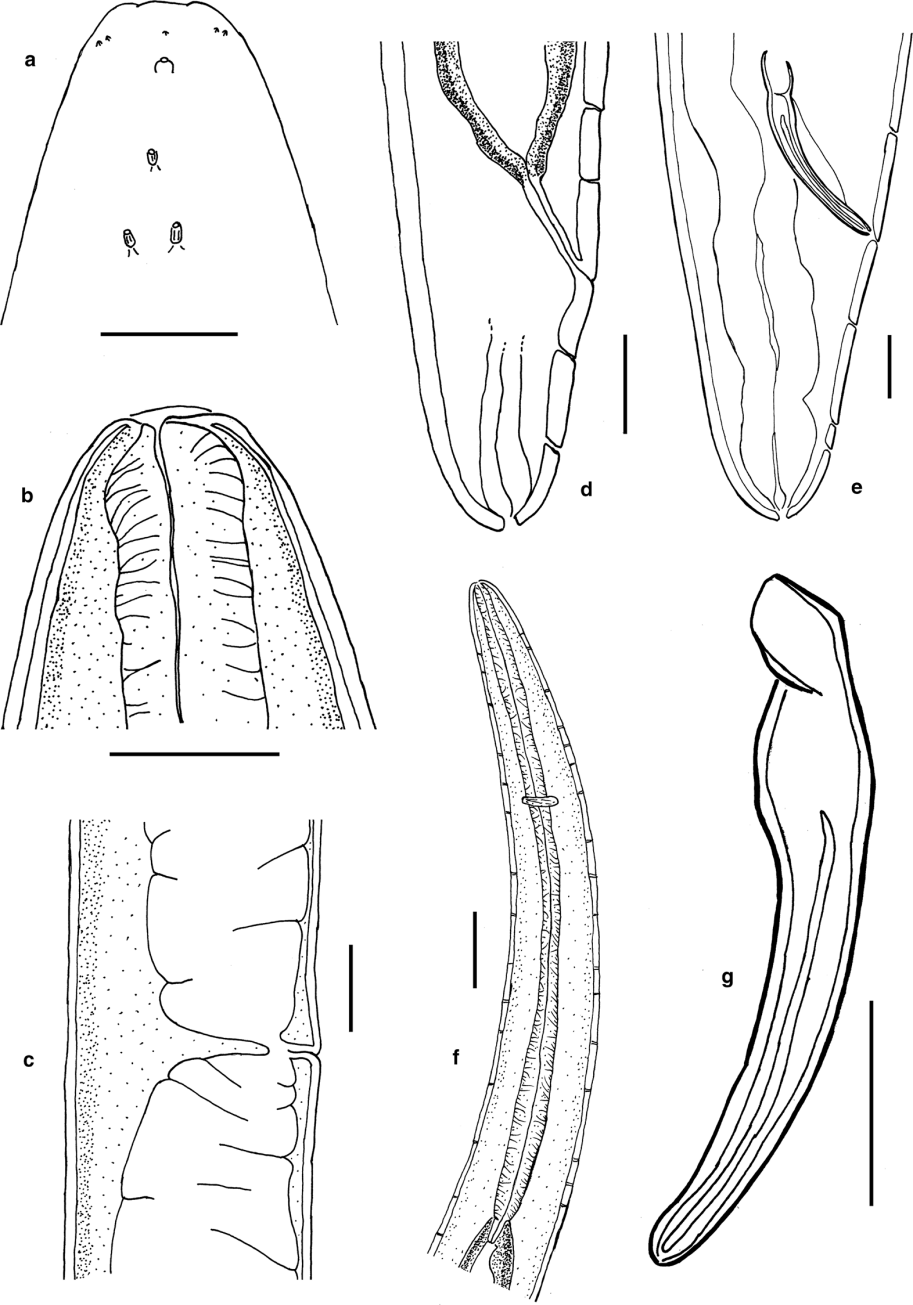
*Aborjinia corallicola* **sp. n.** Female holotype (a–d, f) and males (e, g). a. Anterior end showing outer labial and cephalic sensilla, amphid and body pores; b. Anterior end showing mouth and anterior part of pharynx; c. Vulva and uteri; d. Posterior end with anus, spinneret and ventral row of body pores; e. Male tail; f. Pharynx and nerve ring; g. Spicule. Scale bars: a–b, e, g = 50 µm, c–d = 100 µm, f = 200 µm.

**Fig. 4:**
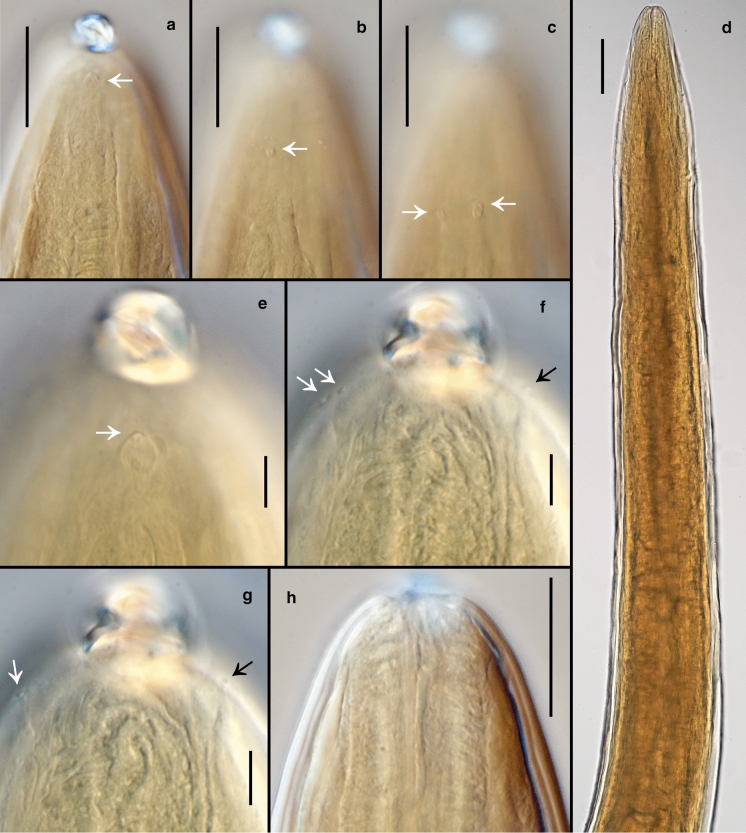
*Aborjinia corallicola* **sp. n.** a. Anterior body end showing amphid (arrow); b. Anteriormost body pore (arrow); c. Second pair of body pores (arrows); d. Pharynx; e. Amphid (arrow); f–g. Outer labial and cephalic sensilla (arrows); h. Anterior part of pharynx. Scale bars: a–c, h = 50 µm, d = 100 µm, e–g = 10 µm.

**Fig. 5:**
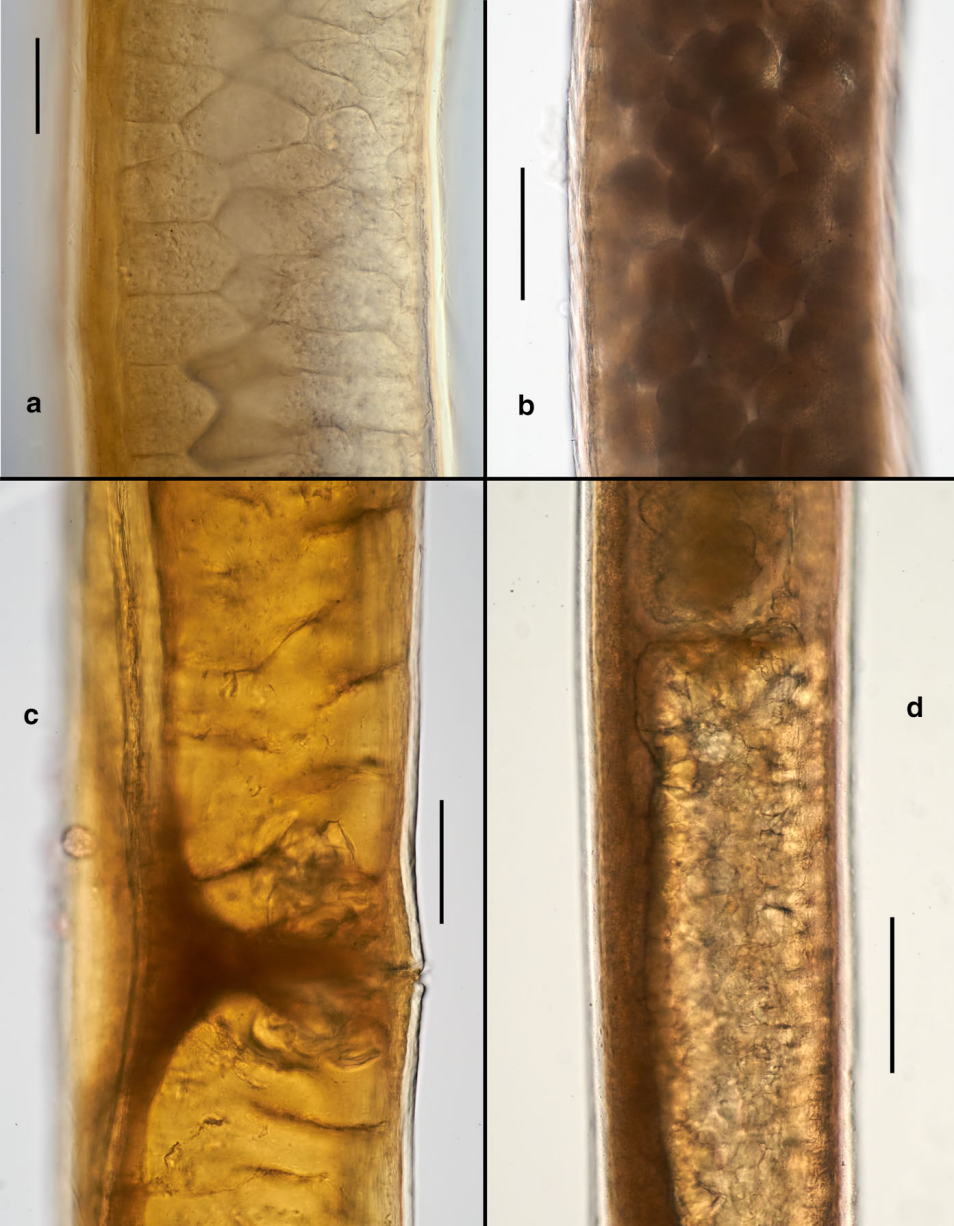
*Aborjinia corallicola* **sp. n.** a. Ovocytes; j. Eggs; k. Vulva and uteri; l. Anterior uterus. Scale bars: a–b = 50 µm, c–d = 200 µm.

**Fig. 6:**
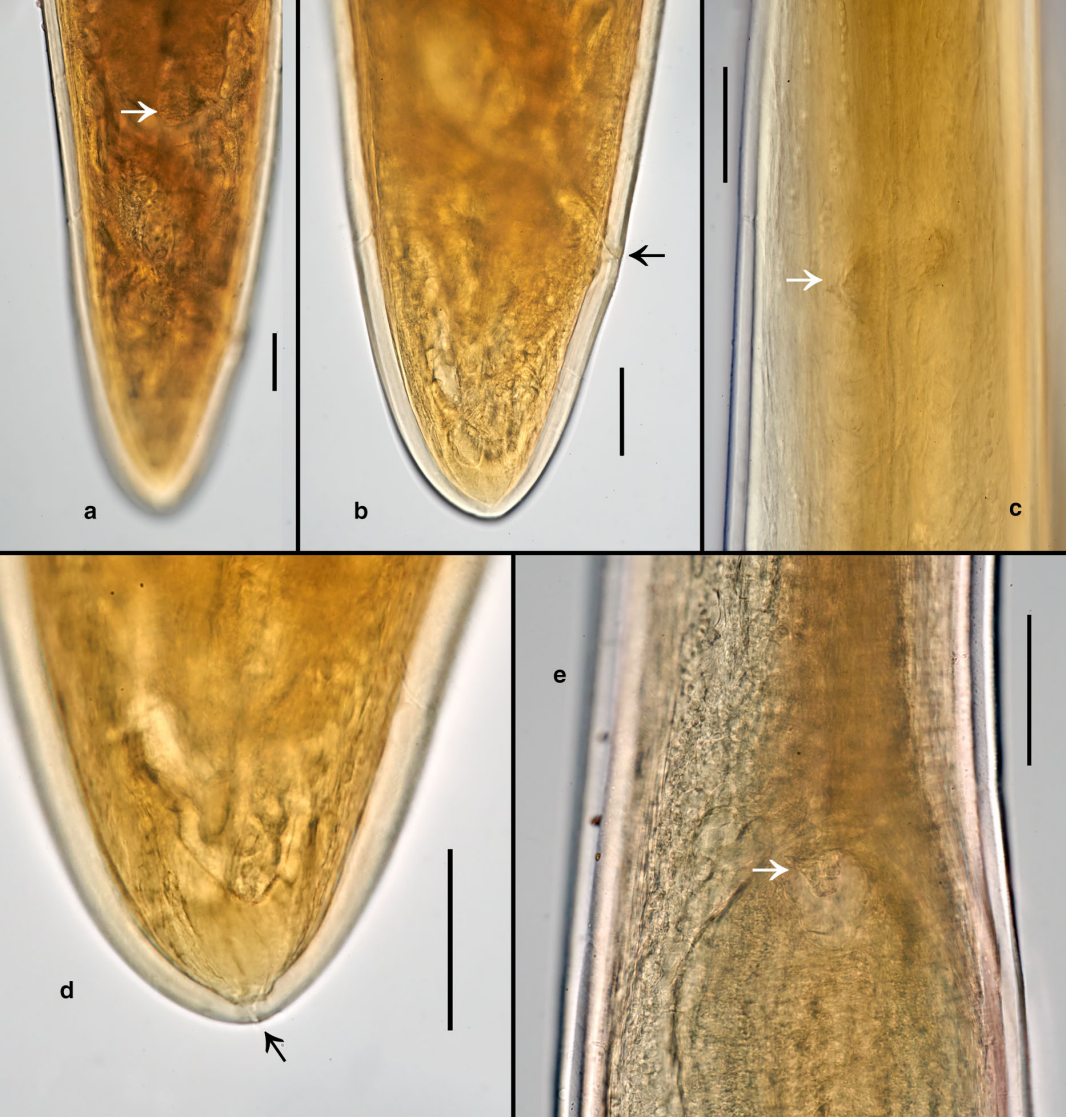
*Aborjinia corallicola* **sp. n.** a. Posterior end of posterior ovary (arrow); b. Rectum (arrow) and tail; c. Nerve ring (arrow); d. Spinneret (arrow); e. Cardia (arrow). Scale bars: a–b, d–e = 100, c = 50 µm.

*Female.* (Figs 3a–d, 3f, 4, 5, 6, Table 2) Body cylindrical, tapering slightly towards both extremities along pharyngeal region and on tail. Cuticle smooth under the light microscope. Body pores distinct, arranged in eight longitudinal rows along the anteriormost part of pharyngeal region (two subdorsal, two subventral and four sublateral); arrangement of body pores along the rest of the body is more irregular. Somatic sensilla indistinct. Cephalic region rounded, continuous with body contour. Lips fused in pairs (dorsal and two ventrosublateral), making a triangular oral opening. Inner labial sensilla not visible. Six outer labial sensilla small papilliform located just in front of cephalic sensilla. Cephalic sensilla small papilliform. Amphideal opening pore-like, located short distance behind the cephalic sensilla, amphidial fovea pouch-like. Buccal cavity very small, simple, without any specific structures. Pharynx uniformly muscularised along its entire length, gradually widening posteriorly but without any valves or bulbs. Cardia small, conoid. Intestinal lumen distinct only in its anteriormost part. Secretory-excretory system and secretory-excretory pore not found. Reproductive system didelphic, amphidelphic. Ovary branches outstretched. Anterior ovary extends anterior but not reaching the cardia. Posterior ovary extends close to anus in mature individuals. Uteri large, tubular. Vagina straight. Vulva located just posterior to midbody, a transverse slit. Tail straight, convex-conoid, with broadly rounded terminus. Caudal glands present, even in adults, opening towards extension via a common spinneret. Spinneret slightly subventral. Candal gland cells located along posterior part of intestine.

**Table 2:** Measurements of *Aborjinia corallicola* **sp. N.**

Sex	Females							males	
Sample	4122-1	4133-16	5846-5	5846-5	11306-1 (holotype)	12180-1	12359-2	12126-2	12180-1
Body length, mm	26.5	33.2	28.5	29.7	25.6	20.8	29.2	17.6	15.5
Vulval / maximal body diameter, µm	442	614	418	438	352	352	338	?	?
Pharynx length, µm	1771	1739	1715	1638	1808	1400	1848	1029	?
Tail length, µm	221	290	211	214	259	176	273	205	242
Anal / cloacal body diameter, µm	221	297	197	221	193	159	148	138	154
A	60	54	68	68	73	59	86	?	?
B	15	19	17	18	14	15	16	17	?
C	120	115	135	139	99	118	107	86	64
c’	1.0	0.98	1.1	0.97	1.3	1.1	1.8	1.5	1.6
V (%)	51.5	53.9	56.5	56.3	53.9	?	52.3	–	–
Outer labial sensilla from anterior end, µm	14	21	14	13	14	16	17	16	17
Cephalic sensilla from anterior end, µm	14	21	14	13	15	17	21	18	21
Amphid from anterior end, µm	31	41	23	27	22	29	28	59	73
Vulva length, µm	52	93	52	79	55	73	66	–	–
Rectum length, µm	121	208	152	138	154	141	162	–	–
Spicules length, µm	–	–	–	–	–	–	–	187	173

*Male.* (Figs 3e & 3g, Table 2) Similar to females in most respects, but internal structures poorly visible due to suboptimal preservation. Amphid is located more posterior than in females and the tail is relatively longer. Male gonads are poorly visible. Spicules paired and symmetrical, weakly arcuate, with round manubrium and fusiform shaft, and thin velum. No pre- or post- cloacal sensilla or supplements.

*Juveniles.* Similar to females in most respects, except for smaller body size and undeveloped reproductive system.

*rRNA.* Sequences include 18 nearly full length and one partial 18S rRNA gene and 19 partial 28S rRNA gene representing D2/D3 domain. Sequence variability of both genes was small, less in D2/D3 domain of 28S (0–2 bases difference) than in 18S (0–10 bases difference) and random.

Relationships

The newly discovered nematode undoubtedly belongs to the family Marimermithidae in having a parasitic lifestyle, small pore-like amphid, muscular and uniformly cylindrical pharynx without developed buccal cavity, intestine not modified into trophosome, paired female gonads with hologonic ovaries and presence of caudal glands. It is placed in the genus *Aborjinia* based on the combination of the following characters: outer labial and cephalic sensilla papilliform in shape and located in one circle, contrary to *Marimermis* (outer labial and cephalic sensilla setiform) and *Ananus* and *Thalassonema* (outer labial and cephalic sensilla in separate circles, if known).

Within the genus *Aborjinia*, the new species is different from *Aborjinia* sp. as described by Miljutin (2003) in a much shorter body (18–42 mm in the new species vs 69–122 mm in *Aborjinia* sp.) and more robust body as seen by a different a-value (54–86 in the new species vs 115–136 in *Aborjinia* sp.) The pharynx is shorter in proportion to the body in *Aborjinia* sp., with the higher b-value (36–37 vs 14–19 in *Aborjinia corallicola* **sp. n.**) and there are also differences in relative tail length in proportion to body length (c-value is 226–364 in *Aborjinia* sp. and 64–139 in *A. corallicola* **sp. n.**). The excretory pore was not found in *A. corallicola* **sp. n.** but it is present in the *Aborjinia* sp. and is located 400 µm from the anterior end. Host species are also different (coral *Acanella arbuscula* in new species vs *polychaete Laetmonice* spp. *Aborjinia* sp.).

From *Aborjinia eulagiscae* the new species differs in much shorter body (14–42 mm in the new species vs 103–132 mm in *A. eulagiscae*), much shorter tail (176–290 µm in the new species vs 850 µm in *A. eulagiscae*), presence of caudal glands and spinneret in adults (vs absent in *A. eulagiscae*), different host species (coral *Acanella arbuscula* in new species vs *polychaete Eulagisca gigantea* in *A. eulagiscae*). The excretory pore is present in *A. eulagiscae* but was not found in the *A. corallicola* **sp. n*.***

The type species of the genus *Thalassonema, Thalassonema ophioctinis*, is similar to *Aborjinia corallicola* **sp. n.** in body size and other measurements. The new species, however, has much longer pharynx 1400–1848 µm, in comparison to 700–1040 µm in *T. ophioctinis*. Otherwise, are they quite similar in size and proportions. This *Thalassonema* lacks caudal glands and spinneret and has an echinoderm as a host (*Ophiocten amnitinum*), while *Aborjinia corallicola* **sp. n.** inhabits a cold-water coral.

*Thalassonema ophiacanthis* known from one male is similar to *Aborjinia corallicola* **sp. n.** in general body size, being only slightly bigger (44 mm vs 14–42 mm in *Aborjinia corallicola* **sp. n.**) with slightly longer tail (310 µm vs 176–290 µm in *Aborjinia corallicola* **sp. n.**) and different b- and c- values (b=27, c=146 vs b=14–19, c=64–139 in *Aborjinia corallicola* **sp. n.**). In *T. ophiacanthis* cephalic sensilla are arranged in separate circles while in *A. corallicola* they are in a single circle. Moreover, the cephalic sensilla in *T. ophiacanthis* are located closer to the anterior end (10 µm) in comparison to 13–21 µm in *A. corallicola* **sp. n.** and the amphid is much further down from the anterior end (140 µm in *T. ophiacanthis*) than in *A. corallicola* **sp. n.** (22–73 µm). Furthermore, the caudal glands are absent in *T. ophiacanthis* and the host is possibly an echinoderm (*Ophiacantha antarctica*) and not a coral.

Single species or the genus *Ananus*, *Ananus asteroideus*, differs from *Aborjinia corallicola* **sp. n.** in much larger body length (90–100 mm vs 14–42 mm in *Aborjinia corallicola* **sp. n.**), and the cephalic sensilla that in this species are placed in separate circles (vs in single circle in *Aborjinia corallicola* **sp. n.**). Moreover, the tail in *Ananus asteroideus* is blunt with mucro in contrast to the tail of *Aborjinia corallicola* which is convex-conoid and broadly rounded. Both anal opening and caudal glands are absent in *Ananus asteroideus* (vs present in *Aborjinia corallicola* **sp. n.**). This species is a parasite of *Diplopteraster perigrina* (Echinodermata).

All three known species of *Marimermis* are distinct from *Aborjinia corallicola* **sp. n.** and all other Marimermithidae in the presence of long setiform outer labial and cephalic sensilla. Moreover, *Marimermis litoralis* differs from *A. corallicola* **sp. n.** in body size and almost every other measurement: body length and a, b and c-value are bigger in *M. litoralis* while the pharynx and the tail are shorter than in *A. corallicola* **sp. n.** (see Table 3). The tail is blunt and devoid of caudal glands and spinneret (convex-conoid with caudal glands and spinneret in *A. corallicola* **sp. n.**). It is unknown what host this species inhabits.

**Table 3:**
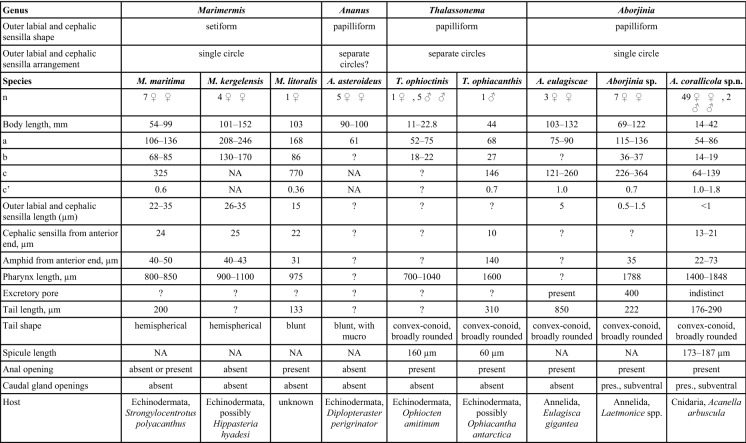
Comparison between different genera and species of the family Marimermithidae

Like the previous species, *Marimermis kergelensis* is bigger in both body length (101–152 mm vs 14–42 in *A. corallicola* **sp. n.**) and has different proportions of the body (a-, b- c-values). The length of the pharynx is also a character that differs between the two species, the pharynx is shorter (900–1100 µm) compared to *A. corallicola* **sp. n.** (1400–1848 µm). The tale is hemispherical and both anal opening and caudal glands are absent (vs present in *A. corallicola* **sp. n.**). The host is possibly a *Hippasteria hyadesi* (Echinodermata).

Even though *Marimermis maritima* has the shortest body length of the *Marimermis* (54–99 mm) they are almost twice as long as the *A. corallicola* but with much shorter pharynx 800–850 (vs 1400–1848 in *A. corallicola* **sp. n.**). The tail is hemispherical and caudal glands are absent (convex-conoid with caudal glands and spinneret in *A. corallicola* **sp. n.**). Like the other *Marimermis*- species, this nematode has Echinodermata as a host, in this case *Strongylocentrotus polyacanthus*.

## Discussion

**Fig. 7:**
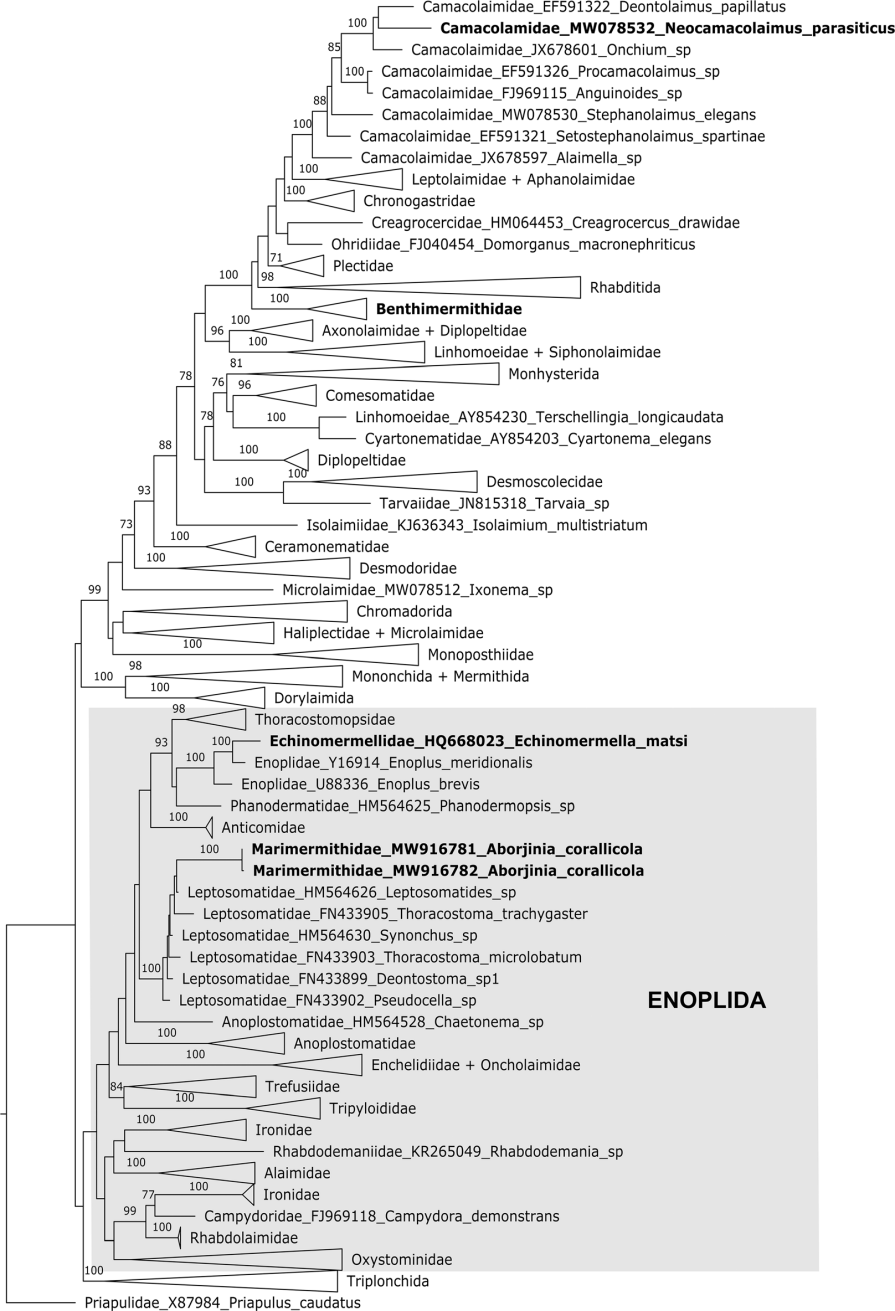
Phylogenetic position of *Aborjinia corallicola* **sp. n.** based on 18S rDNA.

*Phylogenetic position of Marimermithidae:* The results from the phylogenetic analysis based on 18S rRNA gene (Fig. 7) suggest that the closest relatives to the *Aborjinia corallicola* **sp. n.** (family Marimermithidae*)* are the family Leptosomatidae Filipjev, 1916. In fact, the phylogeny suggests that Marimermithidae are nested within Leptosomatidae. It is a group with eight subfamilies and around forty genera, but the only ones included in our study are *Leptosomatides* sp., *Synonchus* sp., *Pseudocella* sp., *Deontostoma* sp., *Thoracostoma microlobatum* Allgén, 1947 and *Thoracostoma trachygaster* Hope, 1967*.* Other species have not been sequenced.

Leptosomatidae is a diverse group of free-living aquatic nematodes found in different marine habitats and a part of the meiobenthic fauna in both shallow waters and in the deep sea (down to 9436 m) in different parts of the world. They are generally quite big for nematodes and some species in this family are considered the biggest free-living nematodes with a length up to 50 mm (Tchesunov, 2006). Even though they are considered free-living, some of the species live their lives in close association with other animals, like the *Leptosomatides marinae* Platonova & Galtsova, 1976 that lives in the cavities in sponges *Halichondria* sp. grooving on rocks in the littoral zone in Akkeshi bay Japan (Kito and Hope, 1999). *Leptosomatum* Bastian, 1865 is another closely related genus that often uses sponges as a host. For example, *Leptosomatum bacillatum* (Eberth, 1863) was found in a sponge *Halichondria panicea* (Pallas) living in the lower littoral zone in the Netherlands and is believed to have a facultative association with this sponge. But the same nematode was found in association with several other species of aquatic organisms in both France and the Netherlands (Bongers, 1984) – not just *Halichondria panicea* as a host but also several different species of algae, such as *Codium* sp., *Polysiphonia* sp., *Cladophora* sp., *Chondrus crispus* Stackhouse and *Laminaria digitata* (Hudson).

The genus *Pseudocella* Filipjev, 1927 includes some of the largest free-living nematodes known. They were often found in the intertidal zones of North America (Hope, 1967) and also among brown algae in the littoral zone at the seas of the Arctic basin (Platonova & Galtsova, 1976). Nematodes in the genus *Deontostoma* can also be considered to be quite large, from 10 mm to 37 mm (Platonova & Galtsova, 1976). Some species from this genus have been found in sediment from holdfasts of *Egregia* sp. collected from intertidal rocks along the coast of California (Hope, 1967), but also occurs among brown algae in the littoral zone in the Arctic, subarctic, Antarctic and subantarctic waters (Platonova & Galtsova, 1976). Among many known species, *Thoracostoma microlobatum* are found in the littoral zone in sediment held by holdfasts of *Egregia* sp. taken from intertidal rocks on the coast of California (Hope, 1967). Closely related species *T. trachygaster* has also been found along the California coastline where they are associated with hold fasts of kelp species such as *Egregia* sp. and *Macrocystis* sp. at a depth of 7 to 20 meters (Derycke et al., 2010).

There are relatively few known species of aquatic nematodes parasitic on invertebrates. What makes this discovery so interesting is that the closest relatives to *Aborjinia corallicola* **sp. n.** are species in the family Leptosomatidae which also includes some symbiotic species (Bongers, 1984; Hope, 1977). Therefore, these two groups, *Aborjinia* and Leptosomatidae form a clade containing species that are symbiotic (including parasitic) on invertebrates, and there is a relatively large gap to the other clades which include parasitic nematodes.

One thing that stands out about *A. corallicola* **sp. n.** is that they live underneath the coral tissue when other species of parasites in this group (Marimermithidae) live in the body cavity of their host. Another peculiarity that remains unexplained is why there are so few marine nematodes adapted to a parasitic lifestyle (excluding parasites from the order Rhabditida, which are secondarily marine) considering the fact that parasitism is such a successful strategy. The pre-adaptations required for symbiosis/parasitism with marine invertebrates are smaller in terms of oxygen availability, osmotic pressure, higher temperature (if it is a warm-blooded host), than for those terrestrial parasites living on land or having a vertebrate host. Specifically, in case of marine nematodes the adaptations required for parasitism would not be so considerable if they already live in the commensalistic association with the prospective host organism, taking shelter on or in it, where the change in oxygen level, osmotic pressure or temperature will be minimal. Little is known about *A. corallicola* **sp. n.** and there are still many unanswered questions, such as whether they live as free-living in some part of their lives. Considering how few species of marine nematodes are known to science, the genus *Aborjinia* and the family Marimermithidae may have close relatives as free living or parasites awaiting to be discovered in the deep of the oceans.

*Relationships between Marimermithidae and Echinomermella:* Nematode parasitism in aquatic invertebrates is quite uncommon, only known in very few lineages but has arisen independently a couple of times over the history of nematodes. The closest parasitic relative to the *A. corallicola* sp. n. are the *Echinomermella matsi* Jones & Hagen, 1987 included in our phylogenetic analysis and its congener *E. grayi* (Gemmill, 1901)*. E. matsi* was found in the pervisceral coelom of the sea urchin *Strongylocentrotus droebachiensis* (O.F. Müller) living among the kelp in the Vestfjorden in northern Norway (Jones & Hagen, 1987), while *E. grayi* is known from *Echinus esculentus* L. Both *Aborjinia* and *Echinomermella* belong to the same order Enoplida but are members of two separate sister clades, one that includes *Aborjinia* and Leptosomatidae and the other including *Echinomermella* and four other families: Enoplidae, Thoracostomopsidae, Phanodermatidae and Anticomidae (Fig. 7)*.* Our phylogeny suggests an independent origin of parasitism within the order Enoplida*.*

*Relationships between Marimermithidae and Benthimermithidae*: Originally, *Marimermis*, the type genus of the family Marimermithidae, and *Trophomera* (= *Benthimermis* Petter, 1980), the type genus of the family Benthimermithidae, were described together and placed in the same family Marimermithidae (Rubtsov & Platonova, 1974). Current molecular data support previously published morphology-based conclusions (Tchesunov, 1997a, 1997b) that these organisms represent two evolutionary independent lineages.

## Data Availability

All studied specimens are deposited in permanent and accessible repositories: Swedish Museum of Natural History, Stockholm, Sweden and the Canadian National Collection of Insects, Arachnids and Nematodes. Sequences are deposited in GenBank.
